# Three versus five intravitreal aflibercept injections as the initial loading phase in the treatment of diabetic macular edema: one-year results

**DOI:** 10.5935/0004-2749.20200078

**Published:** 2024-02-11

**Authors:** Akin Cakir, Burak Erden, Selim Bolukbasi, Ozkan Sever, Sezin Ozdogan Erkul, Ozen Ayranci Osmanbasoglu, Gulderen Karaca, Mustafa Nuri Elcioglu

**Affiliations:** 1 Department of Ophthalmology, University of Health Sciences, Okmeydani Training and Research Hospital, Istanbul, Turkey; 2 Department of Ophthalmology, Namik Kemal University, Tekirdağ, Turkey; 3 Department of Ophthalmology, University of Health Sciences, Istanbul Training and Research Hospital, Istanbul, Turkey

**Keywords:** Diabetic retinopathy, Macular edema, Intravitreal injections, Receptors, vascular endothelial growth factor/administration & dosage, Retinopatia diabética, Edema macular, Injeções in travítreas, Receptores de fatores de crescimento do endotélio vascular/administração & dosagem

## Abstract

**Purpose:**

To compare the efficacy of three initial monthly intravitreal aflibercept injections followed by *pro re nata* (3+PRN) dosing versus five initial monthly intravitreal aflibercept injections followed by *pro re nata* (5+PRN) dosing in patients with diabetic macular edema.

**Methods:**

A total of 60 treatment-naïve patients with macular edema who underwent intravitreal aflibercept injections (2 mg/0.05 mL) with at least one year of follow-up were analyzed in this retrospective and comparative study. The patients were divided into two groups according to the number of intravitreal aflibercept injections administered in the loading phase. The 3+PRN group comprised 27 patients, whereas the 5+PRN group comprised 33 patients. The visual and anatomical outcomes were compared between the two groups at baseline and at 3, 6, 9, and 12 months.

**Results:**

Both 3+PRN and 5+PRN, showed statistically significant improvements in the best-corrected visual acuity and central macular thicknesse throughout the study period (p<0.001 and, p<0.001, respectively). There were no significant differences between the two groups in terms of changes in the best-corrected visual acuity and central macular thickness (p=0.453 and, p=0.784, respectively). The mean number of intravitreal aflibercept injections was significantly greater in the 5+PRN group (6.1 ± 0.8) than in the 3+PRN group (3.9 ± 0.8) (p<0.001).

**Conclusion:**

The 3+PRN and 5+PRN regimens showed similar 12-month visual and anatomical outcomes following treatment with intravitreal aflibercept injections in patients with macular edema.

## Abstract (pt)

**Objetivo:**

Comparar a eficácia de três injeções intravítreas mensais iniciais de aflibercept, seguidas de dosagem de *pro re nata* (3+PRN) versus cinco injeções mensais iniciais intravítreas de aflibercept, seguidas de doses de *pro re nata* (5 + PRN) em pacientes com edema macular diabético.

**Métodos:**

Foram analisados neste estudo retrospectivo e comparativo 60 pacientes que não receberam tratamento prévio com edema macular e foram submetidos a injeções intravítreas de aflibercept (2 mg/0,05 mL) com pelo menos um ano de acompanhamento. Os pacientes foram divididos em dois grupos de acordo com o número de injeções intravítreas de aflibercept administradas na fase inicial. O grupo 3+PRN compreendeu 27 pacientes, enquanto o grupo 5+PRN compreendeu 33 pacientes. Os resultados visuais e anatômicos foram comparados entre os dois grupos no período inicial e aos 3, 6, 9 e 12 meses.

**Resultados:**

Tanto os grupos 3+PRN quanto 5+PRN mostraram melhoras estatisticamente significativas na acuidade visual melhor corrigida e na espessura macular central ao longo do período de estudo (p<0,001 e p <0,001, respectivamente). Não houve diferenças significativas entre os dois grupos em termos de alterações na acuidade visual melhor corrigida e na espessura macular central (p=0,453 e p=0,784, respectivamente). O número médio de injeções intravítreas de aflibercept foi significativamente maior no grupo 5+PRN (6,1 ± 0,8) do que no grupo 3+PRN (3,9 ± 0,8) (p <0,001).

**Conclusão:**

Os regimes 3+PRN e 5+PRN mostraram resultados visuais e anatômicos semelhantes em 12 meses após o tratamento com injeções intravítreas de aflibercept em pacientes com edema macular.

## INTRODUCTION

Diabetic macular edema (DME) is characterized by retinal thickening within the central retina due to failure of the blood-retinal barrier, which causes extensive or focal leakage and retinal edema^(1)^. DME is the leading cause of loss of vision in patients with diabetic retinopathy and is a growing public health concern with increasing prevalence worldwide. In the Wisconsin Epidemiologic Study of Diabetic Retinopathy, it has been reported that 20% of patients with type 1 diabetes and 25% of patients with type 2 diabetes eventually develop DME after 10 years of follow-up^(2)^. However, DME was observed in 27.5% of diabetic patients in a recent study^(3)^.

Several therapeutic options for DME are available, including laser photocoagulation, anti-vascular endothelial growth factor (anti-VEGF) administration, intravitreal steroids, and surgical therapy^(1)^. In the past decade, anti-VEGF therapy has become the mainstay of treatment for center-involved DME after several randomized clinical trials demonstrated its superiority compared to other therapeutic strategies, such as laser therapy and steroids^(4-7)^.

Aflibercept (Eylea^®^; Regeneron Pharmaceuticals, Tarrytown, NY, USA) is a 115 kDa recombinant fusion protein consisting of portions of the extracellular domains of human VEGF receptors 1 and 2, fused to the Fc portion of human immunoglobulin-G1^(8)^. Similar to bevacizumab and ranibizumab, aflibercept binds all isoforms of VEGF-A; however, it also binds VEGF-B and placental growth factors 1 and 2^(9)^. Intravitreal aflibercept injection (IVA) has been approved for the treatment of DME on the basis of the results of the VIVID and VISTA studies^(10,11)^. In these studies, efficacies were compared between regimens consisting of 2 mg of IVA administered every four weeks (2q4) and 2 mg of IVA administered every eight weeks (2q8) after five initial monthly doses; these efficacies were also compared with macular laser photocoagulation. At weeks 52 and 100, IVA demonstrated significant superiority in functional and anatomic results over macular laser photocoagulation, with 2q4 and 2q8 displaying similar efficiency^(10,11)^. However, specific regimens involving an initial loading phase were lacking in the VIVID and VISTA studies; in the DA VINCI Study, a regimen of 2 mg of IVA, administered in three initial monthly doses and then on an as-needed basis (PRN), demonstrated results consistent with those of 2 mg IVA administered every four weeks^(7)^. Moreover, a 13.3-letter gain was achieved within one year in Protocol T with six injections initially followed by PRN dosing^(12)^; this increased letter gain indicated that higher initial doses led to improved outcomes. However, in the VIVID and VISTA studies, the gain was 10.7 letters with a regimen consisting of five initial monthly doses followed by bimonthly injections. No consensus has been established regarding whether all patients require additional initial injections, and no direct comparison of these two initial loading regimens (three versus five) has been performed in a single study.

In this study, we aimed to compare three versus five initial monthly loading doses of 2 mg of IVA, followed by PRN treatment, in terms of mean changes in visual acuity and central macular thickness (CMT) at one year in patients with treatment-naïve DME.

## METHODS

This study was conducted at the Department of Ophthalmology, Okmeydanı Training and Research Hospital, Turkey, and was approved by the Clinical Research Ethics Committee of the institution. The study was carried out in compliance with the recommendations of Good Clinical Practice and the tenets of the Declaration of Helsinki. Written informed consent was obtained from all patients for inclusion in the study.

Patients with center-involved and treatment-naïve DME (secondary to either type 1 or type 2 diabetes mellitus), all of whom had been given IVA (2 mg/0.05 mL) following a PRN regimen between August 1, 2016, and August 30, 2018, were identified in our institutional database. The medical records of these selected patients were retrospectively reviewed and the following patients were excluded from the study: (1) patients who switched from IVA to ranibizumab or intravitreal dexamethasone implant throughout the one-year period and (2) patients with a history of grid laser photocoagulation, vitreoretinal surgery, glaucoma, and/or other concomitant macular/retinal disorders (e.g., retinal vein occlusion or age-related macular degeneration). All patients were required to have a minimum of 12 months of follow-up. Ultimately, 60 patients were eligible for inclusion on the basis of the aforementioned criteria.

The patients were divided into two groups according to the number of IVA doses administered in the loading phase: patients who had received IVA in three consecutive initial monthly doses constituted the 3+PRN group and those who had received IVA in five consecutive initial monthly doses constituted the 5+PRN group. The patients were assigned to the 3+PRN or 5+PRN schemes on the basis of the local regulations of the Medical Enforcement Declaration in Turkey without any defined clinical criteria. All patients were followed up monthly after the loading phase and given additional IVA (PRN regimen) if any of the following retreatment criteria was met: CMT ≥300 mm, any serous macular detachment and/or intraretinal fluid present, an increase of ≥50 mm in CMT compared with previous measurements, and loss of one Snellen line or ≥5 Early Treatment Diabetic Retinopathy Study (ETDRS) letters from the previous best-corrected visual acuity (BCVA).

In this chart review, the following data were collected for all patients: a detailed ophthalmologic examination, fundus fluorescein angiography findings (VISUCAM^®^ 524; Carl Zeiss Meditec, Jena, Germany), and spectral-domain optical coherence tomography (SD-OCT) findings (Spectralis^®^ OCT; Heidelberg Engineering, Heidelberg, Germany). Center-involved DME was defined as DME with CMT ≥300 mm in the central subfield with intraand/or subretinal fluid. In the follow-up, treatment response was monitored by SD-OCT, using the tracking mode of the instrument. Anterior segment biomicroscopy, dilated fundoscopy, and Goldmann applanation tonometry were performed during all visits. Visual acuity was measured using the Snellen and ETDRS charts; the results were converted into logarithm of the minimum angle of resolution (logMAR) units for subsequent statistical analyses.

The main outcome measures were mean changes in the BCVA and CMT recorded throughout the study period. In addition, the following parameters were noted for all patients: intraocular pressure (IOP), total number of IVA doses, duration of diabetes, HbA1c levels, and the presence of any ocular and/or systemic side effects.

All statistical analyses were performed using IBM^®^ SPSS Statistics software (version 25.0, IBM Corp., Armonk, NY, USA). Descriptive analyses were expressed using means and standard deviations for normally distributed variables and medians/percentiles for variables that were not normally distributed. Univariate analyses (interand intragroup comparisons) were performed using either parametric or nonparametric tests. The proportions of patients in the two groups who gained 10 letters or more were compared using Fisher’s exact test. Repeated measures analysis of variance was used to evaluate changes in the BCVA, CMT, and IOP over time; variables were grouped as between-subject factors. Greenhouse-Geisser correction was used when the sphericity assumption was violated. In order to investigate the associations between variables, correlation coefficient and significance values were calculated using Pearson’s test. An overall type I error level of 5% was considered to be statistically significant.

## RESULTS

Twenty-seven patients (18 men, 9 women) were included in the 3+PRN group, whereas 33 patients (14 men, 19 women) were included in the 5+PRN group. The mean age was 58.7 ± 11.7 years for the 3+PRN group and 59.1 ± 9.6 years for the 5+PRN group (p=0.876). Both groups had comparable baseline clinical and demographical characteristics. There was a statistically significant difference in the mean number of IVA doses between the two groups (p < 0.001) (Table 1).

**Table 1:** Baseline demographic and clinical characteristics of the patients in this study

	3+PRN^*^ group n=27	5+PRN group n=33	p-value
Age (years), mean ± SD	58.7 ± 11.7	59.1 ± 9.6	0.876
Sex, *n* (% female)	9 (33.3%)	19 (57.5%)	0.074
Lens status, *n* (% phakic)	24 (88.8%)	27 (81.8%)	0.495
HbA1c, mean ± SD	8.2 ± 1.4	7.8 ± 1.4	0.413
Duration of diabetes (years)	13.4 ± 5.7	14.0 ± 5.0	0.631
Types of diabetes, *n* (% type 2)	25 (92.6%)	32 (97%)	0.583
ETDRS BCVA, mean ± SD	64.0 ± 13.4	60.7 ± 14.9	0.370
CMT (µm), mean ± SD	402.4 ± 119.1	420.6 ± 85.7	0.511
Total number of IVA doses	3.9 ± 0.8	6.1 ± 0.8	<0.001

* *Pro re nata*.

ETDRS= Early treatment diabetic retinopathy study; CMT= central macular thickness; IVA= intravitreal aflibercept; SD= standard deviation.

**Figure 1:**
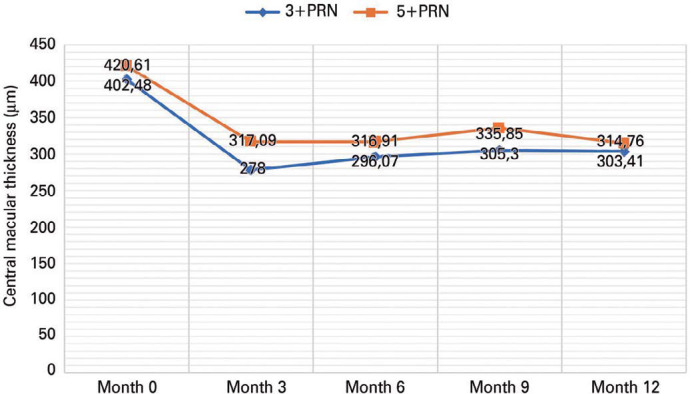
Mean change in CMT from baseline through year 1.

The mean CMT decreased from 402.4 ± 119.1 mm at baseline to 303.4 ± 77.4 mm at month 12 in the 3+PRN group (p<0.001), whereas it decreased from 420.6 ± 85.7 mm at baseline to 314.7 ± 101.1 mm at month 12 in the 5+PRN group (p<0.001). In both groups, the CMT gains with both IVA loading regimens were similar (99.0 ± 123.0 mm versus 105.8 ± 132.6 mm; p=0.784) (Figure 1).

**Figure 2:**
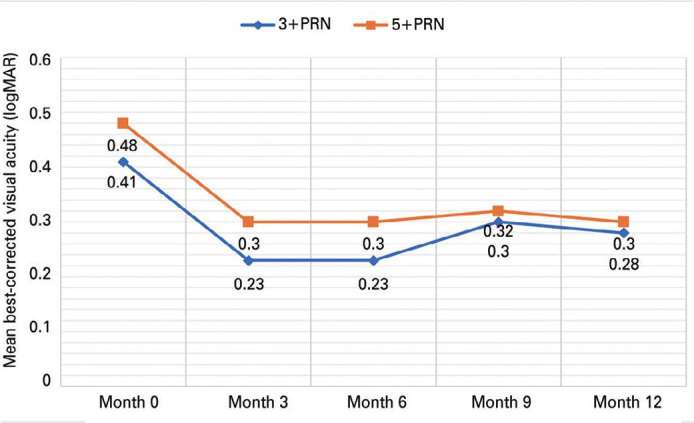
Mean change in BCVA in logMAR from baseline through year 1.

The mean baseline BCVA was 0.41 ± 0.26 logMAR (20/50) in the 3+PRN group and improved to 0.30 ± 0.26 logMAR (20/40) at month 12 (+5.5 letters; p<0.001), whereas it was 0.48 ± 0.29 logMAR (20/50) in the 5+PRN group and improved to 0.28 ± 0.29 logMAR (20/40) at month 12 (+9.8 letters; p<0.001). There was no statistically significant difference between the two groups regarding BCVA improvement (p=0.453) (Figure 2).

**Figure 3:**
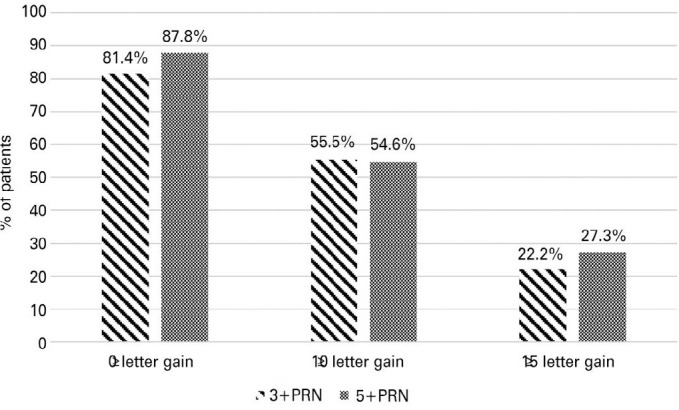
Proportions of eyes that gained _≥_10 and _≥_15 letters from baseline to year 1.

The mean letter gains were 5.5 ± 11.0 in the 3+PRN group and 9.8 ± 18.6 in the 5+PRN group at the end of the follow-up period (p=0.274). The proportions of patients who gained 10 letters or more from the baseline period to month 12 were comparable between the two groups (15 [55.5%] in the 3+PRN group versus 18 [54.6%] in the 5+PRN group; p = 0.729). Although the proportion of patients that gained ≥15 letters from baseline to month 12 was greater in the 5+PRN group, the difference was not statistically significant (6 [22.2%] in the 3+PRN group versus 9 [27.3%] in the 5+PRN group; p=0.729) (Figure 3).

When all patients were assessed as a single group, weak correlations were found between the total number of injections and both BCVA and CMT at month 3 (r=-0.325, p=0.011; r=+0.263, p=0.043; Pearson’s test, respectively).

No significant differences were observed between the two groups regarding IOP changes throughout the study period (p=0.424). None of the patients experienced serious systemic adverse events. The most commonly observed ocular side effects were subconjunctival hemorrhage (21.7%), ocular hyperemia (10%), and vitreous floaters (5%).

## DISCUSSION

This retrospective cohort study demonstrated that both 3+PRN and 5+PRN IVA regimens had similar 12-month visual and anatomical outcomes in the treatment of DME in real-life settings. Although the final acuity in the 5+PRN group was an average of +4.3 letters better compared to the 3+PRN group, this difference was not statistically significant. To the best of our knowledge, this is the first comparison of the 3+PRN and 5+PRN IVA regimens in a single study.

Protocol T, DA VINCI, VIVID, and VISTA were pioneering studies concerning IVA treatment for DME; however, different treatment protocols were used in those trials^(7,10-12)^. The DA VINCI study, which was a phase 2 clinical trial, compared different doses and dosing regimens of IVA versus focal/grid laser photocoagulation. Although the authors stated that the study did not have sufficient power to detect differences between the aflibercept regimens, the “as-needed after three initial monthly doses” (2PRN) group achieved an average gain of 9.7 letters, which was comparable to the monthly dosing group, despite the lower mean number of injections (7.4 injections in the 2PRN group versus 10.8 injections in the 2q4 group) at week 52^(13)^. The one-year results of Protocol T also revealed a 13.3-letter gain with six initial injections, followed by a PRN dosing schedule that resulted in an average of nine to ten injections overall^(12)^.

Although the efficacy of the PRN dosing regimen was demonstrated in the aforementioned studies, there were no arms consisting of a 3+PRN regimen in the VIVID or VISTA studies; in those studies, head-to-head comparisons of 2q4, 2q8 (after five initial loading injections), and macular laser photocoagulation were evaluated^(10)^. In both VIVID and VISTA studies, significant visual improvements were observed with both IVA regimens at weeks 52, 100, and 148; the overall efficacy was similar between the 2q4 and 2q8 IVA groups^(10,11,14)^. The authors of those studies did not clearly explain why they preferred five initial loading injections rather than three initial loading injections^(10)^. We presume that this was because most patients required more than six injections during Protocol T^(12)^; four to six initial injections were used during Protocol I^(6)^. Furthermore, Ziemmsen et al. evaluated treatment responses during the loading phases of VIVID and VISTA and concluded that functional and anatomic improvements continued after the fourth and fifth initial 2q4 injections, suggesting that an intensive and sufficiently long loading phase may be beneficial^(15)^. Conversely, in a recent study, Schwarzer et al. suggested that not all patients with DME required a fixed loading phase when initiating anti-VEGF treatment; this conclusion was based on the results of their investigation of the real-life outcomes of an anti-VEGF treat-and-extend regimen without a fixed loading phase in patients with treatment-naïve DME^(16)^. On the basis of these data, the optimal dosing schedule for the loading phase of IVA in patients with DME remains unclear.

It has been demonstrated that VEGF levels are increased in both the vitreous and aqueous humors of patients with diabetic retinopathy^(17)^. However, the VEGF concentrations might not have been elevated in all patients to the same extent, which may have resulted in different individual responses to the loading phase. Thus, not all patients may require the administration of higher initial injections. An intensive dosing schedule is also controversial in terms of its economical aspects. Regnier et al. reported that the lifetime cost of treating patients with DME in the UK was £20,019 for ranibizumab PRN and £25,859 for a bimonthly aflibercept dosing regimen^(18)^. Therefore, we believe that the implementation of an initial loading dosing schedule tailored to each patient may be a more favorable treatment approach. Although complete resolution of DME might be achieved with three initial injections, continuation of the loading phase with a fourth injection may be useful for determining whether the visual acuity is increasing. Clinicians may choose not to continue with the fifth injection if the patient appears to reach a plateau in letter scores.

In our study, the mean letter gains in both regimens were lower than those previously reported in randomized controlled trials; however, they were consistent with data from other real-world studies^(19,20)^. We presume that this is a result of the lower numbers of injections used in real-life settings. Nevertheless, real-life studies are advantageous in that they more closely resemble daily clinical practice.

Our results revealed that the BCVA gain was better (4.3 letters) for the 5+PRN group at month 12, although this difference was not statistically significant. The higher mean number of injections in the 5+PRN group may have been responsible for this result. There are currently no clearly defined predictors for the identification of patients with DME who would clearly benefit from a more intensive initiation scheme. Consequently, a great number of different initiation schemes have been recommended^(21,22)^. Only the predictors of final BCVA have been studied; for instance, in the Protocol T study, the baseline visual acuity was predictive of visual outcomes^(12)^. However, we presume that it is most important to determine which patients require more injections. Our results indicated that patients who had lower BCVA and higher CMT at month 3 needed more injections overall, regardless of the initiation scheme. Therefore, we suspect that five initial loading injections would be appropriate for patients who respond poorly to three initial injections.

The strength of this pilot study is that it compared these two initial loading regimens in real-life settings. The main limitations of the study include its lack of randomization, retrospective nature, and small sample size. When aflibercept was first approved for DME treatment by the Social Security Institution in Turkey, five initial loading doses were mandatory for a specified period. All patients underwent five initial injections in that period. The Medical Enforcement Declaration was subsequently revised: obligations were repealed, and ophthalmologists were allowed to begin treatment with either three or five initial injections. We have begun performing three initial injections in clinical practice, in accordance with these local regulations. The patients were selected for scheme 3+PRN or 5+PRN according to the aforementioned local regulations, without any predefined criteria. In addition, the baseline BCVA and CMT values of the two groups were comparable, which refutes the hypothesis that patients with greater CMT or worse BCVA initially received five injections. Therefore, we believe that our results were not influenced by a selection bias.

The 3+PRN IVA regimen resulted in visual and anatomical outcomes similar to those of the 5+PRN IVA regimen with a smaller number of injections for the management of patients with treatment-naïve DME. Five initial loading injections may not be necessary for each patient. We speculate that the present real-life data may help emphasize the potential importance of an individua lized loading phase in the treatment of DME. Further prospective, randomized, controlled trials are needed to support and refine our results.
